# Supplementary material for: Splice-Junction-Based Mapping of Alternative Isoforms in the Human Proteome
Source: Cell Rep. Author manuscript; Available in PMC 2020 Jan 15. (PMC6961840; doi:10.1016/j.celrep.2019.11.026)
Supplement: 2 [file NIHMS1546469-supplement-2.zip › DF1/PXD006675/LeftAtrium/LeftAtrium_49_LDB3_VVVNSPAK.pdf]

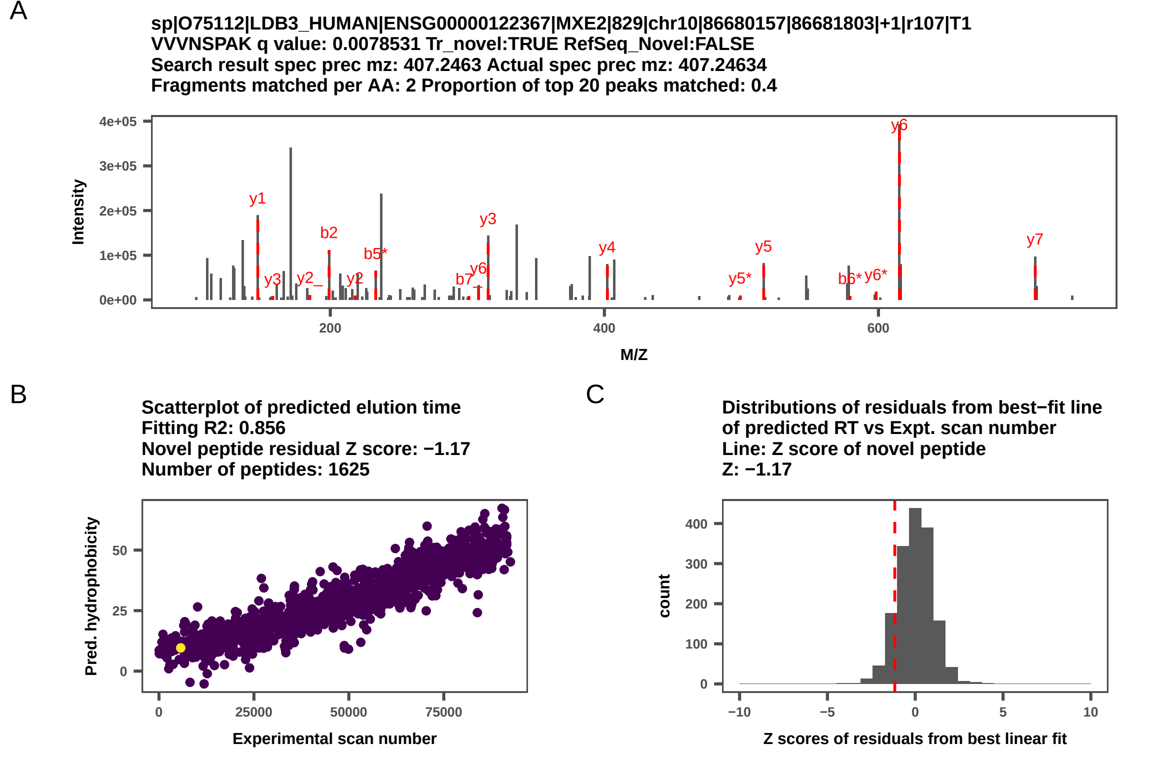
A
sp|O75112|LDB3_HUMAN|ENSG00000122367|MXE2|829|chr10|86680157|86681803|+1|r107|T1
VVVNSPAK q value: 0.0078531 Tr_novel:TRUE RefSeq_Novel:FALSE
Search result spec prec mz: 407.2463 Actual spec prec mz: 407.24634
Fragments matched per AA: 2 Proportion of top 20 peaks matched: 0.4
Intensity
4e+05
3e+05
2e+05
1e+05
0e+00
y1
y3
y2_
y2
b2
b5*
b7
y6
y3
y4
y5*
y5
b6*
y6*
y6
y7
200
400
600
M/Z
B
Scatterplot of predicted elution time
Fitting R2: 0.856
Novel peptide residual Z score: −1.17
Number of peptides: 1625
Pred. hydrophobicity
50
25
0
0
25000
50000
75000
Experimental scan number
C
Distributions of residuals from best−fit line
of predicted RT vs Expt. scan number
Line: Z score of novel peptide
Z: −1.17
count
400
300
200
100
0
−10
−5
0
5
10
Z scores of residuals from best linear fit
